# Strain-induced modulation of temperature characteristics in ferrimagnetic Tb–Fe films

**DOI:** 10.1038/s41598-021-85642-3

**Published:** 2021-03-18

**Authors:** Shinya Ota, Pham Van Thach, Hiroyuki Awano, Akira Ando, Kentaro Toyoki, Yoshinori Kotani, Tetsuya Nakamura, Tomohiro Koyama, Daichi Chiba

**Affiliations:** 1 Institute of Scientific and Industrial Research, Osaka University, Ibaraki, Osaka 567-0047 Japan; 2 Department of Applied Physics, The University of Tokyo, Bunkyo, Tokyo 113-8656 Japan; 3 Toyota Technological Institute, Nagoya, Aichi 468-8511 Japan; 4 Institute of Material Science, Vietnam Academy of Science and Technology, 18-Hoang Quoc Viet, Hanoi, Vietnam; 5 Japan Synchrotron Radiation Research Institute (JASRI), Sayo, Hyogo 679-5198 Japan; 6 Center for Spintronics Research Network at Osaka, Osaka University, Toyonaka, Osaka 560-8531 Japan

**Keywords:** Ferromagnetism, Spintronics

## Abstract

This study investigates the effect of strain on the compensation temperature of ferrimagnetic Tb–Fe films formed on a flexible substrate. The compensation temperature is determined by the anomalous Hall measurement, and an application of 1.2% tensile strain reduces the compensation temperature by 12 K. X-ray magnetic circular dichroism reveals that approximately 5% of Fe magnetic moment and approximately 1% of Tb magnetic moment are reduced by an application of 0.9% tensile strain at the room temperature. To understand the greater reduction in Fe magnetization compared with that in Tb and the compensation temperature reduction simultaneously, a model applying molecular field theory is analyzed. Changes in three types of exchange coupling between Fe and Tb atoms are speculated to be caused by the strain.

## Introduction

A large magnetic anisotropy change has been observed by applying a %-order strain to a flexible substrate on which magnetic films is deposited^1–3^. This is due to the inverse magnetostriction effect and the ability of metallic thin films on flexible-substrates^4^ to tolerate such a large strain. In particular, an alloy of rare-earth (RE) and transition-metal (TM) elements with a large magnetostriction constant shows magnetic anisotropy change on the order of sub-Tesla^2^. Thus far, studies regarding the inverse magnetostriction effect in thin films have focused on magnetic anisotropy.

Alloys between 3*d*-TM and more than half RE elements are ferrimagnetic, where local magnetizations of RE and TM occur with different magnitude anti-parallel couples. Because the temperature dependence of the two magnetization components differ, the total magnetization can be zero even at a temperature below the Curie temperature when the two components cancel out, which is defined as the compensation temperature *T*_comp_. In this study, we investigate the inverse magnetostriction effect on the *T*_comp_ of Tb–Fe samples. We demonstrate that the *T*_comp_ is changed by a strain application, accompanied by magnetic moment changes.

## Results

### Sample preparation

**Figure 1:**
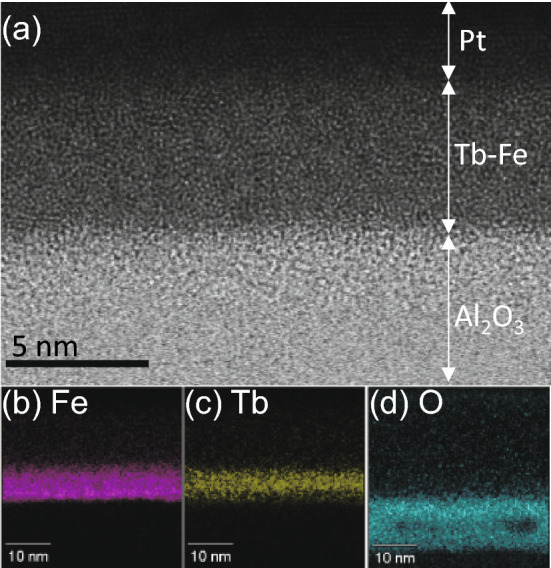
(**a**) Bright field scanning TEM image of our Tb–Fe sample taken before the strain application. Atomic distribution of (**b**) Fe, (**c**) Tb and (**d**) O obtained by EDS.

Tb–Fe samples were prepared on a flexible polyethylene naphthalate (PEN) substrate using dc magnetron sputtering at room temperature. The PEN substrate used in this study was Teonex Q65H (Teijin-Dupont Films Ltd.). In this study, the deposition of Tb–Fe film was performed using two different methods, i.e., co-sputtering and alternative sputtering. We confirmed that in both cases the Tb–Fe layers are the amorphous alloy and have similar magnetic properties. Figure 1 shows the transmission electron microscopy (TEM) image and the energy dispersive X-ray spectroscopy (EDS) of the 6 nm Tb_27_Fe_73_ layer deposited on the substrate. The TEM image has been taken before the strain application. Here, 10-nm-Al_2_O_3_ buffer layer was deposited using atomic layer deposition. 10 nm Pt layers were deposited on the Tb–Fe layers to prevent surface oxidization. The bright field scanning TEM image in Fig. 1a shows no clear separation between Fe and Tb layer and crystal structure. This indicates that the present Tb–Fe layer is amorphous alloy. Figure 1b–d show the atomic distribution obtained by EDS. As shown, the composition in the Tb–Fe layer is not uniform, i.e., Fe is rich and Tb is sparse at the bottom of the Tb–Fe layer. In addition, a slight oxidization at the bottom side of the Tb–Fe layer is observed.

### Method

**Figure 2:**
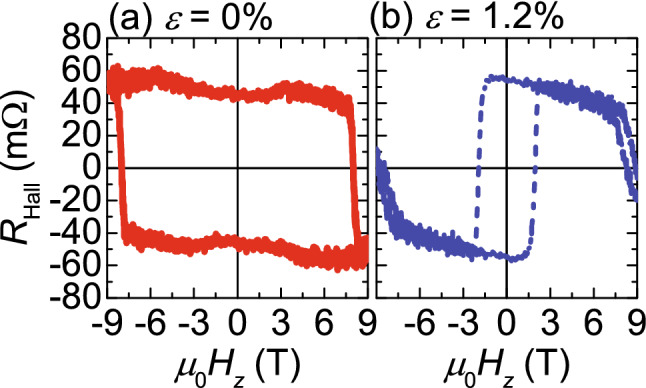
Anomalous Hall resistance curves of the Tb–Fe sample measured at 260 K without and with the strain (*ε*  = 0% and 1.2%, respectively).

The magnetic properties of the Tb–Fe films were measured using the anomalous Hall effect by applying a tensile strain *ε* to the PEN substrate. Figure 2a,b show the anomalous Hall curves for a Tb_32_Fe_68_ sample measured at 260 K (near *T*_comp_) without and with strain (*ε* = 0% and 1.2%, respectively). The vertical axis shows the Hall resistance *R*_Hall_, which was determined form the Hall voltage divided by the current, and an offset voltage was eliminated. For the Hall measurement, the film was defined into a 30-µm-wide Hall bar using photolithography and Ar-ion milling, and Cu wires were bonded using CircuitWorks conductive epoxy (Chemtronics). A direct current of 300 µA was applied, and the Hall voltage was measured. A physical properties measurement system (Quantum Design, Inc.) was used to control the temperature *T* and apply a perpendicular magnetic field *H*_*z*_. A tensile machine made of brass was used to apply *ε* (see Ref.^2,3^ for details). Both ends of the PEN substrate were tightly fixed by grippers and *ε* was applied by increasing the distance between the grippers. *ε* is defined as *ε* =*Δd*/*d*, where *d* and *Δd* are the distance of the grippers before *ε* application and its change.

### Strain modulation of *T*_comp_ measured by the anomalous Hall measurement

In RE–TM films, the anomalous Hall signal reflects the perpendicular magnetization component of the TM because the anomalous Hall coefficient of the TM is known to be positive and dominant, whereas that of RE is negative^5^. Therefore, in general, the sign of *R*_Hall_ is opposite to the net magnetization at low temperatures (*T* < *T*_comp_), in which the magnetization of RE is dominant, and the situation is opposite at high temperatures (*T* > *T*_comp_). In this framework, at *T* = 260 K, the Tb magnetization is dominant before a strain is applied (see Fig. 2a). By contrast, the sign of the anomalous Hall loop is reversed under *ε* = 1.2%, suggesting that the Fe magnetization becomes dominant. This means that the *T*_comp_ is changed by a strain application. Another interesting point is that the strain application decreases the coercivity *H*_c_ by several Tesla (Fig. 2b). The anomaly *R*_Hall_ behavior around ± 8–9 T in Fig. 2b has been previously observed in TbFe film^6^.

**Figure 3:**
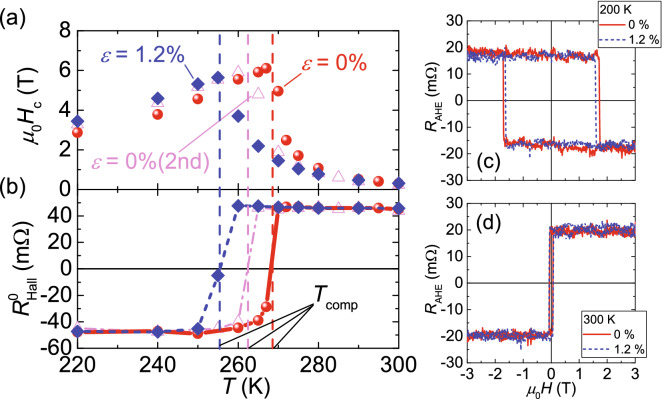
Temperature *T* dependence of (**a**) coercivity *H*_c_ and (**b**) Hall resistance at *μ*_0_*H*_*z*_ = 0 T (${R}_{\mathrm{Hall}}^{0}$). Results of the anomalous Hall measurement performed at (**c**) 200 K and (**d**) 300 K.

**Figure 4:**
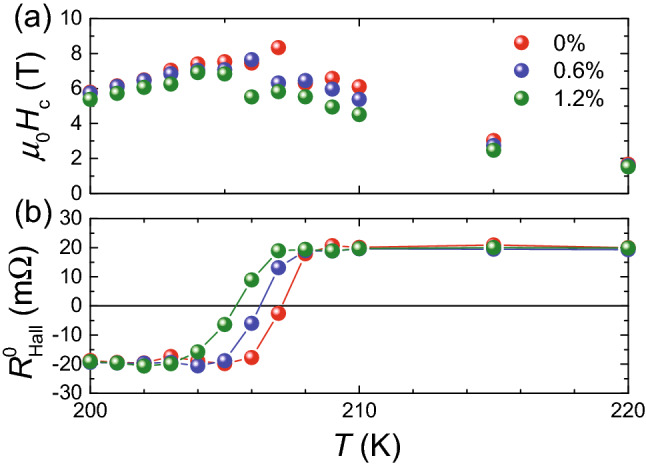
Temperature dependences of the coercivity (**a**) *H*_C_ and (**b**) *R*^0^_Hall_ for *ε* = 0 (red), 0.6 (blue) and 1.2% (green) obtained using Tb/Co ferromagnetic film.

The *T* dependence of the *H*_c_ of the Tb–Fe sample was checked, as shown in Fig. 3a. For double-coercivity curves such as those in Fig. 2b, the smaller coercivity was adopted as the *H*_c_. A *H*_c_ peak is observed at a certain *T*, which corresponds to the *T*_comp_^7,8^. The *R*_Hall_ at *µ*_0_Hz = 0 T (${R}_{\mathrm{Hall}}^{0}$) is shown in Fig. 3b. The sign of ${R}_{\mathrm{Hall}}^{0}$ is defined as the sign of *R*_Hall_ at *µ*_0_*Hz* = 0 T after sweeping back from *µ*_0_*Hz* =  + 9 T. In this study, *T*_comp_ = 268.5 ± 1.5 K for *ε* = 0% was determined as the *T* of the sign reversal in ${R}_{\mathrm{Hall}}^{0}$, which corresponds to the peak of *H*_c_. Figure 3a,b summarize the results of the strain application. The circle, diamond, and triangle points in Fig. 3a,b indicate that strains of *ε* = 0%, 1.2%, and 0% for the second time are applied, respectively. The *H*_c_ and ${R}_{\mathrm{Hall}}^{0}$ plots in Fig. 3a,b show that *T*_comp_ reduces by 12 K with *ε* = 1.2%. There is a partial irreversibility after removing the strain. Figure 3c,d show the results of the anomalous Hall measurement performed at 200 K and 300 K, which are apart from the compensation temperature *T*_comp_. No clear change in the coercivity and/or shape of the magnetization curve has been observed when the strain was applied at these temperatures. This indicates that the strain effect on the magnetic anisotropy is small in the present TbFe film, whereas the significant anisotropy modulation has been shown at a temperature apart from *T*_comp_ in the ferrimangnetic TbFeCo film^2^. The existence of the oxidization layer and/or composition gradient might be the origin of the small effect. Therefore, the significant *H*_c_ change around *T*_comp_ is dominated by the strain modulation of *T*_comp_. We have also checked the strain dependence of *T*_comp_ using another type of ferrimagnet. Here, a Tb/Co multilayer was used and the result is shown in Fig. 4. One can clearly see that the *T*_comp_ decrease with increasing *ε* is also observed in the Tb/Co system. Moreover, monotonic decrease in *T*_comp_ with ε has been confirmed.

### Strain modulation of the Fe and Tb magnetic moments observed using XMCD

**Figure 5:**
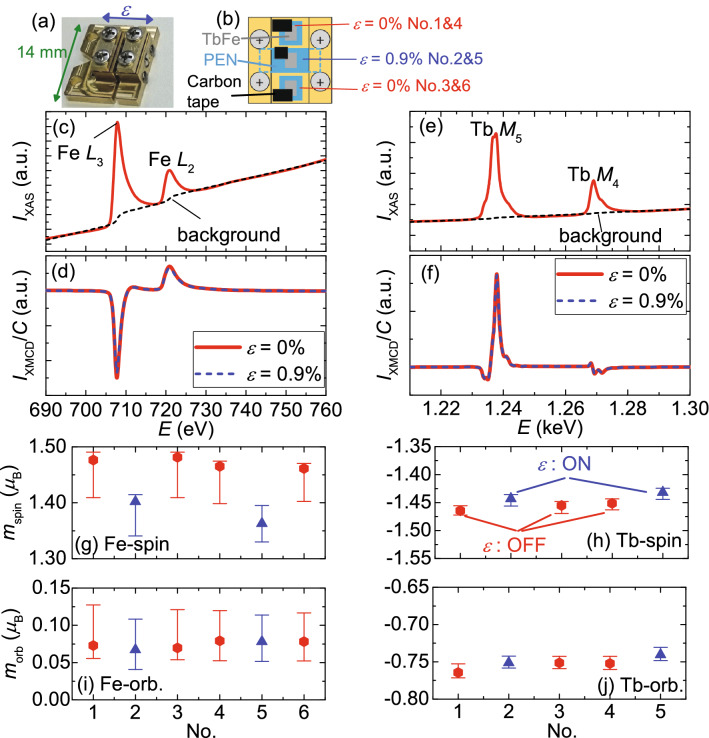
(**a**) Photograph of a tensile machine. (**b**) Schematics of the experimental setup. XAS and normalized XMCD around (**c**,**d**) Fe *L*_2_ and *L*_3_ edges and (**e**,**f**) around Tb *M*_4_ and *M*_5_ edges. (**g**–**j**) *m*_spin_ and *m*_orb_ of Fe and Tb obtained using sum rules.

To investigate the magnetic moment changes induced by the strain atomically, X-ray magnetic circular dichroism (XMCD) was performed using a soft X-ray beamline, BL25SU at SPring-8, at room temperature. The diameter of the X-ray beam spot was ~ 100 µm. The total electron yield method was used to obtain the X-ray absorption spectra (XAS) for positive (*µ*_+_) and negative (*µ*_−_) helicities. The directions of the incident X-ray and external magnetic field were perpendicular to the film plane. The average of the spectra measured under a magnetic field of ± 1.9 T was obtained. For the XMCD measurement, the [Tb(0.44 nm)/Fe(0.31 nm)]_8_ layer deposited on a 10 nm SiN buffer layer was employed. A thinner capping Pt layer of 3 nm was fabricated such that the total electron yield method was applicable. This might have contributed to the lower *T*_comp_ of this sample (~ 70 K) compared with that of the samples discussed above. The XMCD measurement was not performed near *T*_comp_ because to obtain the XMCD spectra for the up and down magnetization states, which are required for the sum rule analysis, is difficult due to the significant increase in the coercivity. The metal layers were defined into a rectangle of 2.0 mm × 1.5 mm and mounted to a sample holder using a miniature tensile machine, as shown in Fig. 5a. Two samples without strain and one with strain *ε* = 0.9 ± 0.2% were inserted to the experiment chamber at once to measure them under the same condition. Carbon tapes were used to establish electric connections and attach the samples. See Fig. 5b for the experimental setup. The three samples were measured twice in the order of the numbers indicated in Fig. 5b.

Figure 5c shows the XAS around the Fe *L*_2_ and *L*_3_ edges, where *I*_XAS_ is the average of two helicities, i.e., *I*_XAS_ = (*µ*_+_  + * µ*_−_)/2. Linear and two-step-like backgrounds (dashed line in Fig. 5c) were eliminated by fitting the pre-edge region (*E* < *x*) and post-edge region (*E* > *y*). Because the selection of *x* and *y* affects the results, we used the best fit condition among various conditions (700 eV < *x* < 706 eV and 732 eV < *y* < 738 eV) for an accurate discussion. Figure 5d shows the XMCD intensity (*I*_XMCD_ = * µ*_+ _− *µ*_−_) normalized by the integration of *I*_XAS_ (*C*) for *ε* = 0% (No. 1) and *ε* = 0.9 ± 0.2% (No. 2). A reduction in |*I*_XMCD_| at the edges due to strain application is observed. Figure 5e,f show the XAS and XMCD spectra around the *M*_4_ and *M*_5_ edges of Tb, respectively. The linear fitting regions for the background subtractions were 1220 eV < *x* < 1230 eV and 1281 eV < *y* < 1287 eV. The strain effect at the Tb edges was not as clear as that of Fe.

The spin magnetic moment *m*_spin_ and orbital magnetic moment *m*_orb_ per Fe or Tb atom was calculated using magneto–optical sum rules^9,10^. The hole numbers for Fe and Tb were assumed to be 3.39^11^ and 6, respectively. The effect of the magnetic dipole was neglected for the Fe case and included as a correction factor of 3/2 for the Tb case according to literature^12^. Figure 5g–j summarize the calculated magnetic moments with respect to the measurement number, where the strains are *ε* = 0% for No. 1, 3, 4, and 6 and *ε* = 0.9 ± 0.2% for No. 2 and 5. The error bars indicate values that can be calculated using different *x* and *y* as indicated above. The larger error bar for Fe case is accompanied with the subtraction of the complicate background signals in XAS spectrum explained above. The moments in Fe and Tb were confirmed to be positive and negative, respectively. It is clear that the *m*_spin_ in Fe, which is a dominant component in the total magnetic moment in Fe, is the most affected by the strain application among the four moments, whereas |*m*_spin_| and |*m*_orb_| in Tb decreased slightly. Although the strain effect is not clear in the raw XMCD spectrum, the slight change in |*m*_spin_| (approximately 1.5%) by the strain application has been confirmed when the sum rule analysis was carefully done. The resultant value of the magnetic moment is sensitive to the background range we have set, which is indicated by the error bar of each data. Nevertheless, the difference in the strain on and off state is statistically significant.

## Discussion

**Figure 6:**
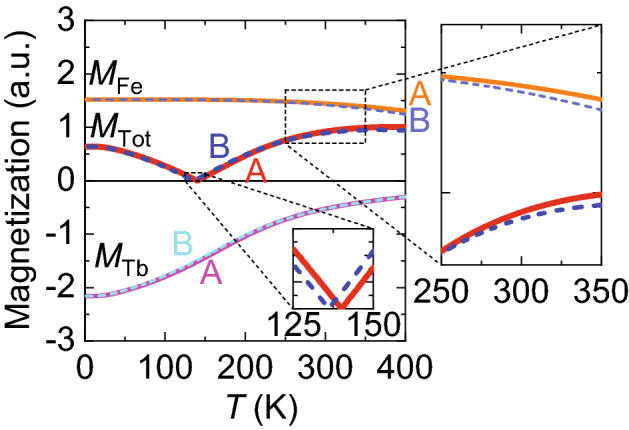
Simulated magnetization dependence on *T* for *M*_Fe_, *M*_Tb_ and *M*_Tot_ = *M*_Fe_ + *M*_Tb_. Parameters used for calculating the curve A are *M*_Fe_(0)/*n*_Fe_ = 2 *µ*_B_, *M*_Tb_(0)/*n*_Tb_ = 9 *µ*_B_, *w*_FeFe_ = 468, *w*_FeTb_ = 12 and *w*_TbTb_ = 28. Parameters *w*_FeFe_, *w*_FeTb_, and *w*_TbTb_ used for curves denoted as B are 8% smaller, 9% larger, and 8% smaller compared with those for A, respectively. Insets are enlarged views around *T*_comp_ and the room temperature.

The decrease in *T*_comp_ (Fig. 3b) with the larger decrease in the Fe moment compared with that of Tb is seemingly inconsistent because *T*_comp_ typically becomes higher if the Fe composition in Tb–Fe is reduced. To demonstrate that such a change can occur, we analyzed a toy model based on molecular field theory that can simulate the temperature dependence of RE–TM systems^13–15^. The molecular fields for the Fe (*H*_Fe_) and Tb (*H*_Tb_) atoms in Tb–Fe can be expressed as

1 $${\mu }_{0}{H}_{\mathrm{Fe}}\left(T\right)={\mu }_{0}{w}_{\mathrm{FeFe}}{M}_{\mathrm{Fe}}\left(T\right)-{\mu }_{0}{w}_{\mathrm{FeTb}}{M}_{\mathrm{Tb}}\left(T\right),$$

2 $${\mu }_{0}{H}_{\mathrm{Tb}}\left(T\right)={\mu }_{0}{w}_{\mathrm{TbTb}}{M}_{\mathrm{Tb}}\left(T\right)-{\mu }_{0}{w}_{\mathrm{FeTb}}{M}_{\mathrm{Fe}}\left(T\right),$$

where *M*_Fe(Tb)_ is the Fe(Tb) component of the magnetization; *w*_FeFe_, *w*_FeTb_, and *w*_TbTb_ are dimensionless quantities that describe Fe–Fe, Fe–Tb, and Tb–Tb magnetic interactions, respectively. The temperature dependence of each magnetization is governed by the Brillouin function *B*_*J*_(*x*):

3 $${M}_{\mathrm{Fe}}\left(T\right)={M}_{\mathrm{Fe}}\left(0\right){B}_{\begin{array}{c}{J}_{\mathrm{Fe}}\end{array}}\left[{M}_{\mathrm{Fe}}\left(0\right){H}_{\mathrm{Fe}}\left(T\right)/{n}_{\mathrm{Fe}}{k}_{\mathrm{B}}T\right],$$

4 $${M}_{\mathrm{Tb}}\left(T\right)={M}_{\mathrm{Tb}}\left(0\right){B}_{\begin{array}{c}{J}_{\mathrm{Tb}}\end{array}}\left[{M}_{\mathrm{Tb}}\left(0\right){H}_{\mathrm{Tb}}\left(T\right)/{n}_{\mathrm{Tb}}{k}_{\mathrm{B}}T\right],$$

where *n*_Fe(Tb)_ is number of Fe(Tb) atoms in a unit volume. In this study, we assumed the angular momenta to be *J*_Fe_ = 1 and *J*_Tb_ = 6^13^. By numerically solving Eqs. (1)–(4), the magnetization–*T* curves of *M*_Fe_, *M*_Tb_, and *M*_Tot_ = *M*_Fe_ + *M*_Tb_ can be obtained. The set of solid curves denoted as A in Fig. 6 were obtained using parameters *M*_Fe_(0), *M*_Tb_(0), *n*_Fe_, *n*_Tb_, *w*_FeFe_, *w*_FeTb_, and *w*_TbTb_, whose compensation temperature was *T*_comp_ = 140 K. We have adopted the calculation result where *T*_comp_ = 140 K, not 70 K, because in this condition all changes in the magnetic properties by the strain (*ΔT*comp < 0, *ΔM*Fe < 0 and *ΔM*Tb < 0) are successfully reproduced. We sought for conditions that suited the experimental results (Δ*T*_comp_ < 0 and Δ*M*_Fe_ < Δ*M*_Tb_ < 0 at *T* = 300 K with the strain application) by changing *w*_FeFe_, *w*_FeTb_, and *w*_TbTb_ and assumed that other parameters were independent of strain. However, no suitable condition was obtained when only one of the three parameters was changed. A few suitable conditions were obtained by reducing *w*_FeFe_ and *w*_TbTb_ and increasing *w*_FeTb_. One of the typical results is displayed by the dashed lines in Fig. 6 (denoted as B). Under this condition, the signs of Δ*T*_comp_, Δ*M*_Fe_, and Δ*M*_Tb_ obtained in the experiments above were well reproduced. The sign of *T*_comp_ change in the simulation is consistent with the experiment, while *T*_comp_ in the simulation is different from that in the AHE measurement. Although we have obtained the magnetic moment of Fe and Tb atoms and its changes by the strain only at room temperature away from *T*_comp_, the simulation successfully reproduces the sign of strain induced *T*_comp_ modulation using these data. Therefore, the modulation of atomic distances by the strain application is expected to yield such changes in the magnetic interactions. For *w*_FeTb_, because the antiferromagnetic coupling is expected to be larger for a larger Fe–Tb atomic distance judging from the RE material dependence^16^, our results appear to be consistent. On the other hand, the changes in *M*_Fe_ and *M*_Tb_ in this simulation (2% and 0.3%) are smaller than the XMCD results (5% and 1.5%). In addition, the situation where *w*_TbTb_ > *w*_FeTb_ is opposite to the previous studies and for *w*_FeFe_, the Bethe curve for amorphous TM materials predicted a larger *w*_FeFe_ for a larger Fe atomic distance^17^, which is contrary to our conclusion. These might suggest that the oxidization or composition gradient, which is not taken into account in the model, also contribute to the strain modulations. The Poisson’s compression, that is, a lateral and in-plane compressions perpendicular to the tensile strain axis might be more important than the tensile strain itself. More experimental and theoretical data are required to fully understand the strain effects.

In conclusion, we investigated the effect of strain on Tb–Fe ferrimagnetic films deposited on a flexible substrate. Changes in coercivity and compensation temperature owing to strain application were clearly observed. The XMCD measurement revealed that the magnetic moment in Fe changed significantly due to strain application at the room temperature. The strain effects can be explained by the modulation in three types of magnetic exchange interactions.

## Data Availability

The data that support the findings of this study are available from the corresponding author upon reasonable request.
